# Challenges of Deep Learning in Cancers

**DOI:** 10.1177/15330338231173495

**Published:** 2023-04-27

**Authors:** Yudong Zhang, Jin Hong

**Affiliations:** 1 School of Computing and Mathematical Sciences, 4488 University of Leicester, Leicester, UK; 2 Brain Information and Human Factors Engineering Laboratory, Zhongshan Institute of Changchun University of Science and Technology, Zhongshan, China

Cancer is a significant crisis worldwide. Firstly, cancer is one of the leading sources of death worldwide. According to World Health Organization, cancer caused estimated 9.6 million deaths in 2018. Secondly, its treatment is costly,^1^ and the corresponding care expenses continue to rise. This puts a weighty financial burden on individuals and families, as well as on healthcare systems and governments. Thirdly, cancer and its treatment drastically impact patients’ quality of life. Treatments such as chemotherapy, radiation therapy,^2^ and surgery cause physical and emotional side effects that are debilitating and long-lasting. Fourthly, not all cancer types are preventable. Many cases are connected to lifestyle factors, for example, smoking, alcohol consumption,^3^ poor diet, and so on. Finally, cancer research is a chief focus of public health efforts around the world. Advances in cancer diagnosis, drug treatment, and disease prevention lead to better survival rates, but there is much research to be studied about its pathology and treatment.

Cancer screening,^4^ diagnosis, prediction, survival rate estimation,^5^ treatment, and control measures are still the foremost challenges in the recent decade. With the development of biomedical imaging, inspection, and health management technologies, medical big data (MBD), such as biomedical images, omics,^6^ and clinical electronic medical records, are accumulating rapidly. How can we use this MBD to build better health records and more accurate prediction models to help disease diagnose and treat cancer better? In the past decade, the rapid development of machine learning (ML) methods has provided many successful cases to answer this fundamental question.

Commonly used ML methods for cancer analysis include linear regression, logistic regression,^7^ decision trees, random forests, support vector machines,^8^ neural networks, k-means clustering, principal component analysis,^9^ naïve Bayes, gradient boosting,^10^ and so on. However, ML methods suffer several shortcomings. Traditional ML models cannot predict outcomes with a high level of accuracy due to a lack of complexity in their underlying algorithms. Scalability is another shortcoming. The number of input variables in traditional ML methods is often limited, making it difficult to build systems that are robust and scalable.

Recently, deep learning (DL)^11^ has been the hottest ML method for cancer analysis nowadays. The reasons are 5 aspects. (i) DL can handle large and complex datasets with many variables, which is essential for massive amounts of genomic and clinical data. (ii) DL can detect patterns and identify correlations in cancer data that might not be visible to the human eye. (iii) DL can help predict how individual patients will respond to different treatments based on their genetic profile, medical history,^12^ and other factors. (iv) DL models can be trained on large cancer datasets, such as mammograms^13^ or 3-dimensional computed tomography scans, to detect early signs of cancer that are invisible to the human eye. (v) DL can predict the effectiveness of new cancer drugs^14^ and identify potential drug targets based on genetic and other data.

Some top DL algorithms include convolutional neural networks, recurrent neural networks, autoencoders, generative adversarial networks,^15^ long short-term memory networks,^16^ capsule networks, and so on. There are many other variants of DL algorithms and combinations of these algorithms for various applications of cancer analysis.^17^

Although ML and DL have shown great potential in helping cancer screening, diagnosis, prognosis, and treatment, many significant data-related problems still hinder the application of ML and DL in cancer analysis. These problems include excessive noise, labeled data deficiency, heterogeneous data, unbalanced data, multisource domain data,^18^ and data isolation.

Interpretability and validation are 2 other issues. Most ML and DL models are often referred to as black boxes because it is difficult to interpret how they arrive at their predictions. In cancer analysis, interpretability is crucial to understanding how the ML and DL models arrive at their decisions and gaining insights into the disease's biological mechanism. Meanwhile, ML and DL models can suffer from overfitting, where the model learns to perform well on the training data but performs poorly on new, unseen data. Valuing ML and DL models in cancer analysis is challenging when the data is limited or biased.

To better apply ML and DL models to cancer analysis, it is important to develop novel data preprocessing^19^ methods, data representation learning methods,^20^ and novel efficient ML and DL models.
